# Recurrent Metastatic Choriocarcinoma Responsive to Etoposide and Cisplatin with Etoposide, Methotrexate, and Dactinomycin: A Case Report

**DOI:** 10.31729/jnma.7081

**Published:** 2022-05-31

**Authors:** Sunil Kumar Das, Arun Shahi, Ram Chandra Panthi

**Affiliations:** 1 Department of General Practice and Emergency Medicine, Patan Academy of Health Sciences, Lagankhel, Lalitpur, Nepal; 2 Department of Oncology, Patan Academy of Health Sciences, Lagankhel, Lalitpur, Nepal; 3 Department of Internal Medicine, Patan Academy of Health Sciences, Lagankhel, Lalitpur, Nepal

**Keywords:** *chemotherapy*, *choriocarcinoma*, *metastasis*, *recurrence*

## Abstract

Choriocarcinoma is a malignant trophoblastic tumour usually of placental origin. It is characterized by early metastasis to the brain and lungs. With early detection, it has a better prognosis with treatment. We report a case of 18 years female at 26 weeks of gestation in her third pregnancy who had a history of treatment for metastatic gestational trophoblastic neoplasm with chemotherapy and radiotherapy two years back. Therefore, she was managed as a case of recurrent choriocarcinoma with brain metastasis with chemotherapy (etoposide and cisplatin with etoposide, methotrexate, and dactinomycin) and was responsive. Her symptoms resolved and |3-human chorionic gonadotropin dropped to normal value (<2.39 mIU/ml) which has shown that timely diagnosis and management can be vital for the successful treatment of brain metastasis.

## INTRODUCTION

Choriocarcinoma is a malignant trophoblastic tumour with gestational or germ cell origin.^1^ Gestational Trophoblastic Disease (GTD) may be non-neoplastic or neoplastic. Gestational neoplasia includes invasive mole, choriocarcinoma, Placental Site Trophoblastic Tumour (PSTT), or Epithelioid Trophoblastic Tumour (ETT). It develops from an abnormal trophoblastic population undergoing hyperplasia and anaplasia, following a molar pregnancy.^2^ For patients with choriocarcinoma, β-Human Chorionic Gonadotropin (β-hCG) measurement can identify progressive lesions and can be used in screening.^3^ Here we reported a case of recurrent choriocarcinoma with brain metastasis that was responsive to chemotherapy with an Etoposide and Cisplatin with Etoposide, Methotrexate, and Dactinomycin (EMA/EP) regimen.

## CASE REPORT

An 18-year-female, married for 3 years who was in her third pregnancy with a history of two abortions presented at the Emergency Department on the 26^th^ Week of Gestation (WOG) with a complaint of tingling sensation in the left upper and left lower limb for 5 days and one episode of focal onset to bilateral tonic-clonic seizure which lasted for about 3 minutes. At the presentation, her level of consciousness on the Glasgow Coma Scale (GCS) was 15/15. Vitals were within the normal limits. Examination of the central nervous system, cardiovascular system, and respiratory system was unremarkable. On the per abdomen, examination showed the uterus to be 24 weeks in size. Per speculum examination showed that her cervix was healthy and no discharge was present on per vaginal examination, cervical os was closed, bilateral adnexa was free, and cervical motion tenderness was absent. Her β-hCG level was 22296.6 mIU/ml. Ultrasonography (USG) (Maternal Scan) showed a singleton pregnancy with a live foetus at 24 weeks of gestation. Magnetic Resonance Imaging (MRI) of the brain was also done which showed an isointense lesion with a hypoechoic rim and mild perilesional edema in the right parietal lobe suggestive of active hemorrhagic metastasis. With this, the patient was then admitted to the oncology ward of the tertiary centre.

Her family history was remarkable. Regarding her past history, 2 years back she had a history of molar pregnancy during her second pregnancy at 6 weeks of gestation, findings of which were supported by histological examination. Contrast Enhanced Computed Tomography (CECT) of the head and chest showed pulmonary and cerebral metastasis at that time. She was treated as a case of choriocarcinoma with brain and lung metastasis for which she received four cycles of chemotherapy and 13 cycles of radiotherapy to the head and abdomen. On subsequent follow up her β-hCG was normal (<2.39 mlU/ml). A repeat Computed Tomography (CT) scan of the head and chest showed regressive lesions compared to the previous imaging modality. Later she was lost to follow up due to the COVID-19 pandemic.

In a view of the current and the past history, the patient was diagnosed with Gravida 3 Abortion 2 at 26+^6^ WOG with high-risk Gestational Trophoblastic Neoplasia (GTN) with brain metastasis. EP-EMA (Etoposide and Cisplatin with Etoposide, Methotrexate, and Dactinomycin) regimen was started for 6 cycles. The patient gave birth to a preterm live foetus via vaginal delivery and during the course, her chemotherapy was continued as per the regimen. Her β-hCG showed a gradual decline and was back to normal (<2.39 mIU/ ml) after 52 days of treatment. On the subsequent follow-up, the patient remained asymptomatic and her β-hCG was monitored which was within normal limits.

## DISCUSSION

Choriocarcinoma is a highly malignant epithelial tumour arising from any trophoblastic tissue (molar pregnancy, abortion, ectopic, preterm/term intrauterine pregnancy). Choriocarcinoma is a malignant gestational trophoblastic disease, which commonly (50%) occurs after a hydatidiform mole. It can occur after a spontaneous abortion in up to 25% of cases and after a normal pregnancy in 22.5%.^1^ Estimates from studies conducted in North America, Australia, New Zealand, and Europe have shown the incidence of the hydatidiform mole to range from 0.57-1.1 per 1000 pregnancies, whereas studies in Southeast Asia and Japan have suggested an incidence as high as 2.0 per 1000 pregnancies.^2^ Choriocarcinoma is known to be sensitive to chemotherapy. Remission rates of up to 70% are reported for patients with metastatic disease.^4^ Metastatic choriocarcinoma of the brain is a curable lesion.^5^

Histologically, choriocarcinoma consists of sheets of anaplastic cytotrophoblasts and syncytiotrophoblasts without chorionic villi, while some trophoblasts, which are intermediate in appearance, may also be seen, the biphasic pattern of obviously malignant appearing mononuclear (cytotrophoblasts) and multinuclear cells (syncytiotrophoblasts) is essentially pathognomonic of choriocarcinoma.^6^ The choice of chemotherapy treatment is based on the combination of the Federation Internationale de Gynecologie et d'Obstetrique (FIGO) anatomic staging and the World Health Organisation (WHO) prognostic scoring system based on risk factors.^7^ According to this scoring system, tumours are classified into two categories: low-risk GTN, if the score is ≤6 and FIGO Stage I-III; and high-risk, if the score is ≥7 regardless of stage and in all cases of FIGO Stage IV disease. The score is associated with the risk of developing chemoresistance to singleagent chemotherapy, and thus guides the choice of first-line chemotherapy.^7^

A common cause of mortality in choriocarcinoma is brain metastasis.^8^ EMA/EP regimen could induce complete remission in 88% of patients with high-risk GTN.^9^ Cases have been reported in different gestational weeks at diagnosis that resulted in metastasis to the lungs, brain, vagina, liver, etc with the delivery being normal vaginal delivery or Caesarean section.^10-13^

Gestational trophoblastic neoplasia is a highly chemosensitive gynaecological malignancy. The metastatic choriocarcinoma was almost fatal prior to the development of effective chemotherapy. With the use of sensitive hCG and highly effective chemotherapy, there is a high chance of remission, often cure.
